# Distinct Roles for Inhibition in Spatial and Temporal Tuning of Local Edge Detectors in the Rabbit Retina

**DOI:** 10.1371/journal.pone.0088560

**Published:** 2014-02-21

**Authors:** Sowmya Venkataramani, Michiel Van Wyk, Ilya Buldyrev, Benjamin Sivyer, David I. Vaney, W. Rowland Taylor

**Affiliations:** 1 Department of Ophthalmology, Oregon Health and Science University, Portland, Oregon, United States of America; 2 Institute of Animal Pathology, University of Bern, Bern, Switzerland; 3 Australian Research Council Centre of Excellence in Vision Science, Queensland Brain Institute, The University of Queensland, Brisbane, Australia; Dalhousie University, Canada

## Abstract

This paper examines the role of inhibition in generating the receptive-field properties of local edge detector (LED) ganglion cells in the rabbit retina. We confirm that the feed-forward inhibition is largely glycinergic but, contrary to a recent report, our data demonstrate that the glycinergic inhibition contributes to temporal tuning for the OFF and ON inputs to the LEDs by delaying the onset of spiking; this delay was more pronounced for the ON inputs (∼340 ms) than the OFF inputs (∼12 ms). Blocking glycinergic transmission reduced the delay to spike onset and increased the responses to flickering stimuli at high frequencies. Analysis of the synaptic conductances indicates that glycinergic amacrine cells affect temporal tuning through both postsynaptic inhibition of the LEDs and presynaptic modulation of the bipolar cells that drive the LEDs. The results also confirm that presynaptic GABAergic transmission contributes significantly to the concentric surround antagonism in LEDs; however, unlike presumed LEDs in the mouse retina, the surround is only partly generated by spiking amacrine cells.

## Introduction

Local edge detectors (LEDs) are small-field ganglion cells that display relatively long latency (sluggish), sustained responses at the onset and termination of a spot illuminating the centre of the receptive field (RF). LEDs have been described in a range of species [1]–[6]. In the rabbit, LEDs have the potential, due to their size and density, to provide a high-acuity representation of the visual world, albeit ambiguous regarding the sign of the local contrast edge. Previous work indicated that the inhibition that produces the antagonistic surrounds of LEDs is mediated largely presynaptically [7] by GABAergic inhibition of bipolar cell terminals [8]. These studies also showed that LEDs receive direct feed-forward inhibition; however, the physiological role of this input remains uncertain.

Both GABA and glycine are fast-acting inhibitory transmitters that gate closely related chloride-permeable channels. It is unclear why these amino-acid transmitters are differentially distributed in the CNS, with GABA mediating most of the inhibition in the brain and glycine mediating most of the inhibition in the spinal cord [9]. Uniquely within the CNS, the retina contains about equal numbers of GABAergic and glycinergic interneurons, termed amacrine cells. Retinal amacrine cells are morphologically and neurochemically diverse, comprising at least 30 distinct types of neurons [10]. GABAergic amacrine cells have wide dendritic fields and usually stratify narrowly within the inner plexiform layer (IPL), in either the ON or OFF sublamina. By contrast, glycinergic amacrine cells have narrow dendritic fields and may branch diffusely through the IPL, encompassing both the ON and OFF sublaminae [11], [12].

Amacrine cells receive excitatory input from bipolar cells and provide inhibitory output both to the dendrites of ganglion cells and to processes of other amacrine cells, as well as back to the terminals of bipolar cells [13]–[15]. The diverse receptive-field properties of ganglion cells are due in large part to the activity of the amacrine cells, which can modulate the activity of ganglion cells both postsynaptically, by inhibiting the ganglion cells directly (feed-forward inhibition), and presynaptically, by inhibiting the bipolar and amacrine cells that provide the synaptic drive (feedback inhibition). Early studies used GABAergic and glycinergic antagonists to establish critical roles for amacrine cell activity in generating the different spiking response properties of ganglion cells [16]. For example, direction-selective ganglion cells and orientation-selective ganglion cells lose their stimulus specificity in the presence of a GABAergic antagonist [16]–[19]. The functional roles of glycinergic amacrine cells, which are more numerous than GABAergic cells, remain poorly understood.

Currently the only glycinergic amacrine cell that has been well characterized is the AII amacrine cell, which mediates rod signalling under scotopic conditions, and also provides excitatory (dis-inhibitory) inputs to OFF-alpha ganglion cells in mouse and rabbit [20]–[22]. However, recent work has demonstrated that other glycinergic amacrine cells contribute to the centre responses of orientation-selective ganglion cells [19], ON and OFF brisk-sustained ganglion cells [23], [24] and LEDs [7], [25]. Much remains unknown regarding the functional roles of glycinergic amacrine cells, but recent work has indicated that glycinergic inputs can modify contrast gain and temporal response properties [23], [24], contribute excitatory drive through dis-inhibition [19]–[22], and even play a role in complex feature detection like orientation-selectivity [19].

Three reports concluded that feed-forward inhibition strongly modulated the temporal response properties of LEDs, by suppressing spiking during rapid global luminance shifts, as will occur when an animal moves its head or eyes [6]–[8], a finding that seemed compatible with the suggestion that inhibition might generate sluggish responses by increasing spike latency [7]. However, a role for inhibition in modulating spike latency was not confirmed in the later report [8], which proposed an additional functional role for glycinergic inhibition, which was to suppress GABAergic surround signals that are activated within the centre of the receptive field. A central goal of this study was to re-examine the role of glycinergic inputs to LEDs.

## Materials and Methods

### Ethics Statement

Experiments were performed on adult pigmented rabbits of either sex. Experimental procedures were in accordance with the National Institutes of Health guidelines for animal use and the protocols were approved by the Institutional Animal Care and Use Committee at OHSU.

The methods for whole-cell recording of the visually evoked currents in retinal ganglion cells have been described in detail previously [24], [26]. The rabbits were dark-adapted for at least an hour and, following sodium pentobarbital overdose, the eyes were enucleated under dim-red illumination and the retinas removed. A piece of inferior retina was placed in a recording chamber perfused with Ames medium at a rate of 5 ml/min and the tissue was maintained at 34–36°C (pH 7.4). The ganglion cells were targeted for recording using infrared differential-interference-contrast (IR-DIC) optics.

Extracellular and patch electrodes were pulled from borosilicate glass to a final resistance of 5–8 MΩ. The extracellular electrodes were filled with Ames medium while the patch electrodes contained: 135 mM Cs-methylsulfonate, 6 mM CsCl, 2 mM Na-ATP, 1 mM Na-GTP, 1 mM EGTA, 2 mM MgCl, 5 mM Na-HEPES and 5 mM QX-314-bromide or chloride. All reagents were obtained from Sigma-Aldrich (St Louis, MO) unless otherwise indicated. Cesium was used in place of potassium to block voltage-gated potassium currents and thereby improve the quality of the voltage clamp at positive potentials. The QX-314 was included to block voltage-gated sodium channels and abolished all spiking activity within 1–2 min of establishing the whole-cell configuration. A liquid junction potential of –10 mV was subtracted from all voltages. Series resistance compensation was not generally applied during recordings. The calculated chloride reversal potential, *E_Cl_*, was about –68 mV when using QX-314-Cl, and –54 mV when using QX-314-Br, assuming that Br is 1.5 times more permeable than Cl through the chloride channels [27]. For current clamp recordings, the patch electrode contained: 128 mM K-methanesulphonate, 10 mM Na-HEPES, 0.1 mM EGTA, 6 mM KCl, 2.5 mM phosphocreatine disodium salt, 2 mM Mg-ATP, 0.3 mM Na-GTP.

The conductance analysis methods have been described previously [7], [19], [26], [28]. Briefly, the light responses were recorded at holding potentials from –110 to +25 mV in 15 mV steps. Total membrane current-voltage relations (I-V) were generated at a reference time-point just prior to light stimulation (leak I-V relation) and at 10 ms intervals during the light stimulus. For each I-V relation, the membrane potential was corrected for the series resistance error, and these voltage-corrected I-V relations were interpolated using cubic spline interpolation and resampled at 9 fixed voltages [19]. Series resistance was measured from the peak of the capacitive transient at the onset of a hyperpolarizing voltage pulse (31±15 MΩ, n = 17). At each time-point, the leak I-V relation was subtracted to obtain the net light-evoked I-V, and a line was fitted to this I-V over the linear range, usually between –85 and –25 mV. The slope and the voltage axis intercept of the light-evoked I-V provided measures of the total light-evoked synaptic conductance (*G_T_*) and the synaptic reversal potential (*V_r_*), respectively. The excitatory component, *G_e_*, and the inhibitory component, *G_i_*, of the synaptic conductance were calculated from *G_T_* and *V_r_*, using an excitatory reversal potential, *V_e_* = 0 mV, and an inhibitory reversal potential, *V_i_* = *E_Cl_*. The excitatory and inhibitory components were calculated as: *G_e_(t) = G_T_(t)(V_r_(t) – V_i_)/(V_e_ – V_i_)*, and *G_i_ (t) = G_T_(t)(V_r_(t) – V_e_)/(V_i_ – V_e_)*.

Flash stimuli were step-modulated spots, centred on the receptive field, whose intensity was increased (bright spot) or decreased (dark spot) from the background level. Flicker stimuli were sinusoidal or square-wave modulated spots centred on the receptive field. All stimuli were generated on CRT computer monitors at refresh rates of 60 or 85 Hz, using only the green gun of the CRT. The stimuli were projected through the microscope and focused onto the photoreceptor outer segments, via the 20× water-immersion objective (N.A. = 0.95). The background light intensity (*L*_BACK_) was set to 150 µW/m^2^ at the retinal surface, which for the green phosphor of the stimulus monitor corresponds to ∼400 photons/µm^2^/s. Assuming a collecting area for the rabbit rods of ∼1 µm^2^, the background intensity was well above the scotopic range. The stimulus light intensity (*L*_STIM_) was set to 30 µW/m^2^ for dark stimuli and to 270 µW/m^2^ for bright stimuli. Thus, the percentage stimulus contrast, defined as C = 100×(*L*_STIM_–*L*_BACK_)*/L*_BACK_, ranged from –80% to +80%. For experiments involving strychnine, a 10× higher background light intensity was used to rule out the rod pathway contribution. The concentration of strychnine used, 0.5 µM, was low enough to obviate non-specific effects due to suppression of α-7 nicotinic acetylcholine receptors [29]. In some experiments, ginkgolide (1 mM), a glycine receptor channel blocker [30], was added to the intracellular solution to attenuate inhibitory inputs.

Measurements were compared using a two-tailed, paired T-test, assuming normal distributions. Differences are noted as significant for P-values less than 0.05. Unless otherwise noted, measurements are quoted as the mean ± standard error of the mean.

## Results

### Glycinergic Inhibition Shapes Temporal Tuning

**Figure 1:**
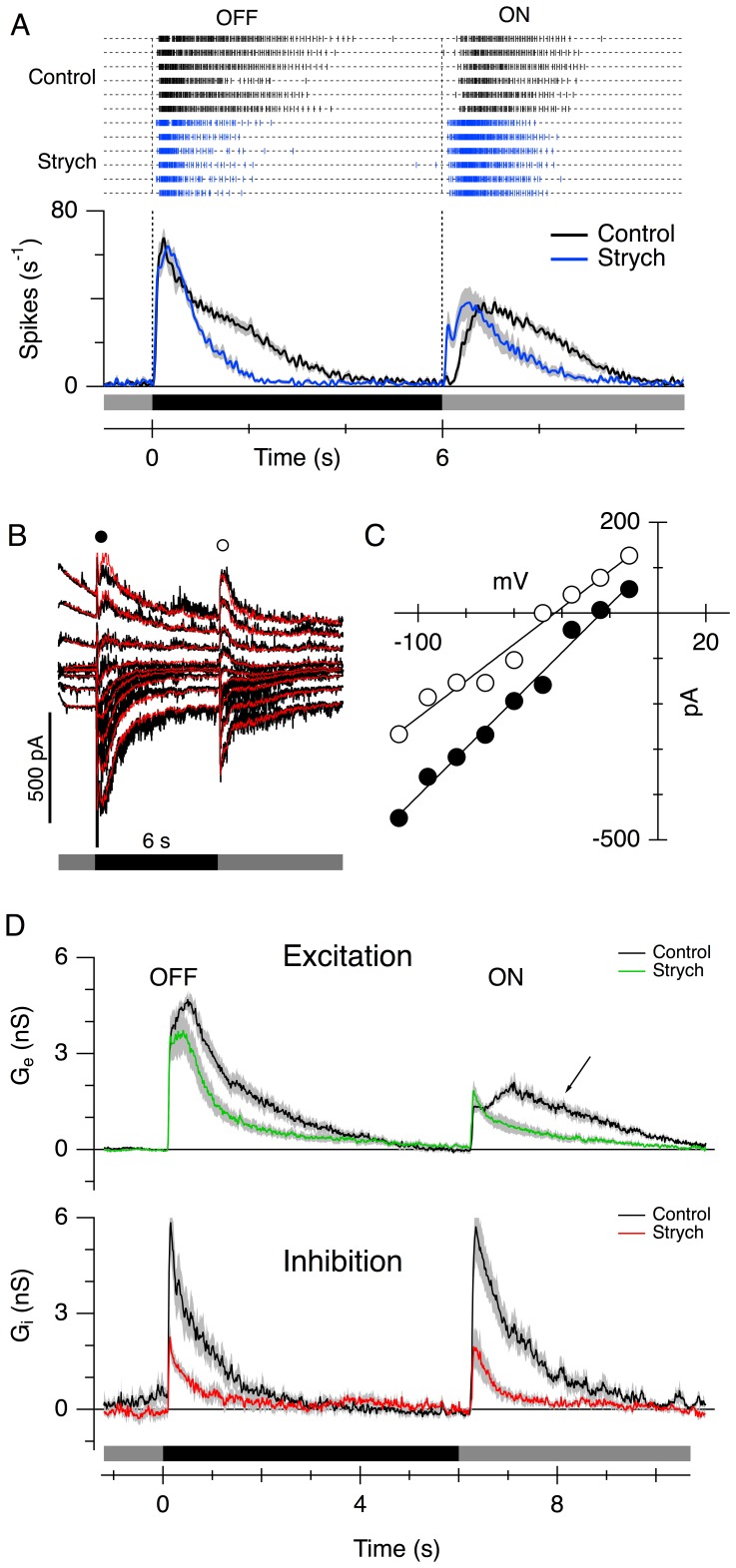
Effects of strychnine on the centre responses of LEDs. (A) The upper panel shows representative spike-raster plots of spike times for 6 consecutive responses to a small dark spot (100 µm diameter) flashed in the centre of the LED’s receptive field for 6 s, in control (black) and with 0.5 µM strychnine (blue); the lower panel shows the spike-time histograms averaged from 8 cells in control (black line) and during application of strychnine (blue line). The stimulus timing is shown by the shaded bars below the traces. The shading shows the standard error of the mean (SEM). (B) Example light-evoked membrane current recorded during stimulation of an LED at holding potentials from –110 to +25 mV in 15 mV steps; each trace shows the response to a single stimulus. The membrane potential was stepped to a new holding potential 2 s before the light stimulus. The stimulus timing is shown by the bars below the traces. The red traces show the current traces calculated from a linear combination of excitatory and inhibitory conductance components. The average of these components for 8 cells is shown in D. (C) Example current-voltage relations of the net light-evoked synaptic currents sampled at two time-points indicated by the filled (OFF) and open (ON) circles in B. The data points were sampled at equally spaced intervals between −108 and −12 mV from interpolations of series-resistance corrected I-V relations. The lines illustrate the linear regressions used to obtain the total synaptic conductance, *G_T_*, and synaptic reversal potential, *V_r_* at each time-point. The magnitude of the excitatory and inhibitory conductances at each time-point were calculated from these values (see Methods). The calculated conductances accurately reproduced the recorded currents, as shown by the red traces in B. (D) Light-evoked synaptic conductance calculated from voltage-clamp experiments during a dark-spot stimulus as for A. The upper panel shows the excitatory conductance (G_e_) averaged from 8 cells in control (black trace) and during application of 0.5 µM strychnine (green trace). The lower panel shows the average inhibitory conductance G_i_ in the same 8 cells in control (black trace) and in the presence of 0.5 µM strychnine (red trace). Strychnine suppressed the peak amplitude of the OFF inhibition by 62±10% (P = 0.0020), and the ON inhibition by 65±6% (P<0.001). The shading shows the SEM.

LEDs respond with sustained firing at the initiation and termination of a step-change of contrast in the receptive-field centre; for a dark-spot stimulus, these responses correspond to OFF and ON responses, respectively (Fig. 1A, upper panel, control traces). Spike onset latency was measured as the time, relative to a contrast transition, at which the spike-rate reached half the peak value. Consistent with previous results [7], the latency to spike onset for the OFF responses was shorter than that for the ON responses (OFF latency = 95±10 ms, ON latency = 491±56 ms, n = 8). When the glycinergic antagonist strychnine (0.5 µM) was added to the perfusate, the delay before spike onset was significantly reduced (OFF latency = 80±9 ms, P = 0.011, ON latency = 142±51 ms, P<0.001). On average, strychnine reduced the spike onset latency by 15±4 ms for the OFF responses, and 349±48 ms for the ON response. The large change in latency for the ON response is clearly apparent in the average spike-time histograms for the 8 cells (Fig. 1A). Moreover, the duration of spike discharge was reduced for both the OFF and ON responses. For the OFF response, the width at half peak of the spike-time histograms was reduced from 1.29±0.16 s in control to 0.79±0.06 s in the presence of strychnine (P = 0.029). Similarly, the ON response was reduced from 1.94±0.14 s in control to 1.02±0.12 s in strychnine (P = 0.0046).

The changes in spike latency indicate that glycinergic transmission contributes to generating sluggish responses, particularly for the ON response. We next sought to determine whether these changes were consistent with the underlying synaptic conductances. The light-evoked synaptic excitation and inhibition were estimated from current responses obtained at a range of membrane potentials (Fig. 1B,C; see Methods). The calculated excitatory and inhibitory components could accurately account for the light-evoked current responses, as illustrated by the superimposed red lines in the example shown in Figure 1B. In control, activation of the excitatory OFF conductance was characterized by an initial rapid increase within the first ∼120 ms, followed by a slower secondary rise to a peak about 400 ms after stimulus onset (Fig. 1D). The delay and initial rapid rise of the ON excitation were similar to those of the OFF excitation; however, the secondary rise was slower for the ON excitation, reaching a peak about 850 ms after the stimulus change. By contrast, both the OFF and ON inhibitory inputs reached a peak within the first ∼120 ms, tracking the rapid rise of the excitation (Fig. 1D). Unlike the excitation, the inhibition declined monotonically after the peak. Moreover, the inhibition was more transient than the excitation, as evident from the widths at half peak amplitude. Half-widths for OFF excitation were longer than for inhibition, (1.11±0.21 s versus 0.51±0.11 s, n = 8, P = 0.047). Similarly, half-widths for ON excitation were longer than for inhibition (2.32±0.30 s versus 0.82±0.10 s, P = 0.043).

These results can be summarized as follows. 1) Feed-forward inhibition activates as rapidly as excitation but is more transient, and hence will tend to contribute to the spike latencies in the LEDs by suppressing early spiking. 2) The duration of the spiking during a steady stimulus is determined largely by the kinetics of the excitatory inputs; this second point is evident from the finding that strychnine made the spiking responses more transient (Fig. 1A), even though the magnitude of the feed-forward inhibition was reduced (Fig. 1D, lower panel), presumably due to the reduced duration of excitation (Fig. 1D). 3) Presynaptic glycinergic mechanisms prolong excitatory inputs to LEDs. This is evident from the reduced amplitude of excitation late in the response during application of strychnine (Fig. 1D). For both the OFF and ON responses, the excitatory amplitude 120 ms after stimulus onset was unaffected by strychnine (the amplitude in the presence of strychnine as a percentage of control was 112±28%, P = 0.89, for the OFF response, and 139±20%, P = 0.063, for the ON response), but became significantly suppressed 1 s after stimulus onset (strychnine suppressed the OFF excitation by 52±9%, P<0.001, and the ON excitation by 50±18%, P = 0.023). These results suggest that, under control conditions, glycinergic transmission suppresses presynaptic inhibition of bipolar cells [8], presumably through serial connections between amacrine cells, resulting in more sustained OFF and ON EPSCs in the LED.

**Figure 2:**
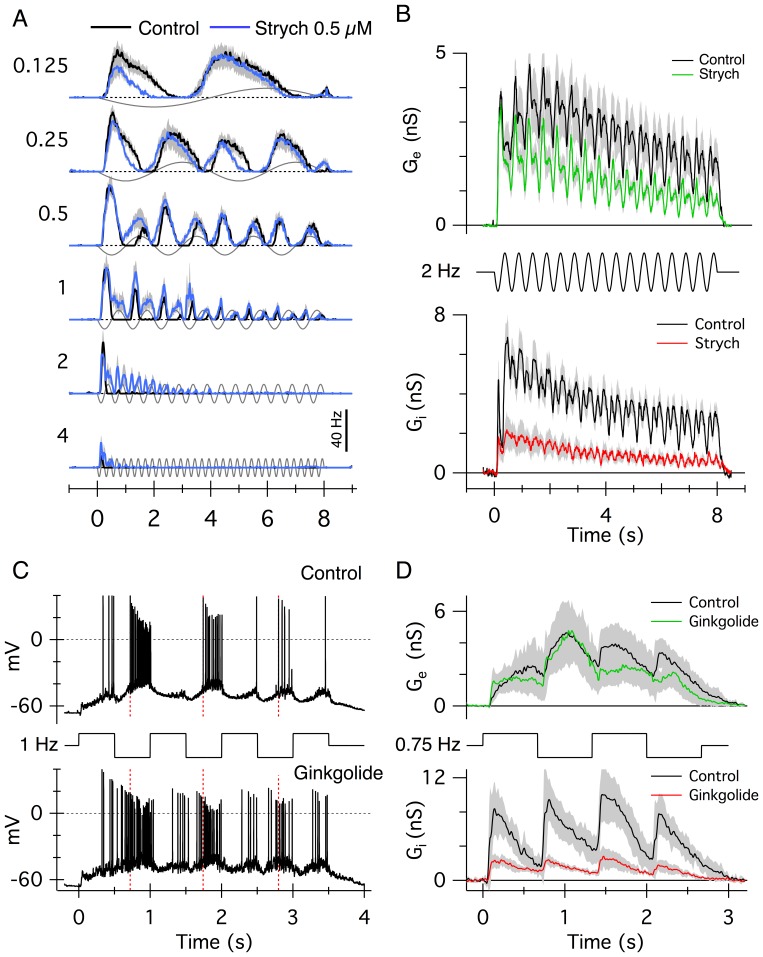
Glycinergic inputs limit the temporal responses of LEDs. (A) Average spike-time histograms generated from extracellular spike responses in 4 LEDs in control (black) and with 0.5 µM strychnine (blue). A significant increase in spiking is particularly evident at frequencies above 0.5 Hz (P<0.05). The stimulus was a 100 µm diameter spot that was sine-wave modulated from –80% to +80% contrast. The stimulus timing is shown in grey; the numbers adjacent to the records indicate the temporal frequency in Hz. (B) Upper panel: average light-evoked excitatory synaptic conductances calculated during 2 Hz stimulation with a small spot, in control (black) and with 0.5 µM strychnine (green). Lower panel: average inhibitory conductance G_i_ in control (black) and with 0.5 µM strychnine (red) during 2 Hz flicker stimulus. The timing of the 2 Hz stimulus timing is shown between the panels. Data averaged from 8 cells, shading shows the SEM. Suppression was not significant for excitation (P = 0.072), but was significant for inhibition (P = 0.0020). (C) Current clamp recordings made from an LED using a potassium-based intracellular solution (see Methods). The lower panel shows the same cell repatched with the addition of 1 mM ginkgolide to the internal solution. The stimulus was a 100 µm spot that was modulated at 1 Hz square wave flicker (timing shown between panels). The red vertical lines show the time of the first spike for the OFF phase of the stimulus in control. Note the shorter spike delay in the presence of ginkgolide (lower panel). (D) Average excitatory conductances (upper panel) and inhibitory conductances (lower panel) in control (black trace) and with 1 mM ginkgolide (colored traces) during 0.75 Hz square wave flicker. Average traces from two groups of 5 cells each are shown. The stimulus timing is shown between the panels. (n = 5, shading shows the standard deviation for each group of cells).

For both the ON and OFF responses, the results indicate that glycinergic pathways shift the temporal responses of the LEDs to lower temporal frequencies. The role of feed-forward inhibition in temporal tuning is also evident from responses during sinusoidal flicker stimulation. At low frequencies (<0.5 Hz), LEDs respond during positive and negative phases of the stimulus cycle as expected for ON/OFF cells (Fig. 2A) but, at higher frequencies, frequency-doubled responses became weaker, presumably due to the limited bandwidth of the ON pathway [7]. This is consistent with earlier reports that the LEDs are essentially OFF-centre cells when their spike responses are probed with flickering stimuli [7], [31], [32]. At frequencies above ∼1 Hz the cell only responds transiently at the onset of the flicker but, in the presence of strychnine, responses are significantly prolonged and continue for several stimulus cycles (Fig. 2A). Analysis of the conductances elicited by a 2 Hz stimulus in 8 cells (Fig. 2B), reveals two factors contributing to the spiking behaviour. 1) Excitation is largest at the onset of the stimulation and adapts during the stimulus (Fig. 2B upper panel, amplitude of excitation in the first stimulus cycle was greater than for the last, P<0.001), and 2) inhibition reaches maximal levels after the first stimulus cycle and is larger than excitation thereafter (Fig. 2B). In the presence of strychnine, the inhibition was more strongly suppressed than the excitation, which appears consistent with the effects on spiking at 2 Hz illustrated in Fig. 2A. Note that the spiking in the presence of strychnine declines during 2 Hz stimulation, consistent with the adaptation of the excitatory inputs seen in the presence of strychnine (Fig. 2B, green).

The data are consistent with the notion that glycinergic mechanisms modulate spiking in LEDs both by direct inhibition and by presynaptic modulation of excitatory inputs. In order to confirm that postsynaptic inhibition was a significant factor in determining spike latencies, we performed current-clamp recordings from LEDs, using K-methanesulphonate-based intracellular solution (see Methods) and recorded spikes in response to a 1 Hz square-wave flicker of a 100µm diameter spot flashed in the centre of the receptive field. The delay to the onset of spiking for the OFF response is shown by the red vertical time-markers in Fig 2C (upper panel). The same cell was then repatched with a new intracellular solution containing 1 mM Ginkgolide B, which blocks the glycine-receptor chloride channel [30] (Fig. 2 C, lower panel). During the same stimulus, the spike latencies for the OFF responses became shorter as the drug diffused throughout the cell and suppressed inhibitory inputs (Fig. 2C, lower panel). Moreover, the ON responses, which were weak in this cell in control conditions, became stronger. Measurements of the excitatory and inhibitory conductances in two groups of 5 cells each, demonstrated that, while the amplitude of the excitation was the same (Fig. 2D, upper panel, P = 0.82), the amplitude of the inhibition was strongly suppressed relative to excitation when ginkgolide was included in the intracellular solution (Fig. 2D, lower panel, P = 0.0074). The fractional suppression of the inhibition was the same for intracellular ginkgolide and bath-applied strychnine shown in Fig. 2B. These results support the notion that postsynaptic inhibition plays a role in determining the temporal response properties of LEDs.

### GABAergic Inhibition Mediates Spatial Tuning

It has been shown previously that surround stimulation of LEDs strongly suppresses the centre responses by reducing both the excitatory inputs [7], [8] and inhibitory inputs [7], presumably reflecting presynaptic inhibition of the bipolar cell terminals that provide direct excitation to both the LEDs and the amacrine cells presynaptic to the LEDs. Significantly, the surround suppression of feed-forward inhibition represents dis-inhibition, and thus will tend to enhance excitability of the LEDs. Therefore, the suppression of spiking responses produced by surround activity must be attributed largely to the reduction in the excitatory inputs, consistent with a surround generated largely by presynaptic inhibition [7]. The OFF surround of LEDs is 4–5× wider than the receptive-field centre [7], [8], suggesting that it is mediated by wide-field GABAergic amacrine cells. Previous work indicated that both GABA_A_ and GABA_C_ receptors mediate surround suppression of excitation [8]. In this study we wanted to determine how surround activation affected feed-forward glycinergic inhibition, and whether spiking amacrine cells contributed to surrounds in LEDs. As a first step, we measured area-response functions for spike responses to confirm the sensitivity of LED surrounds to GABA antagonists under our recording conditions.

**Figure 3:**
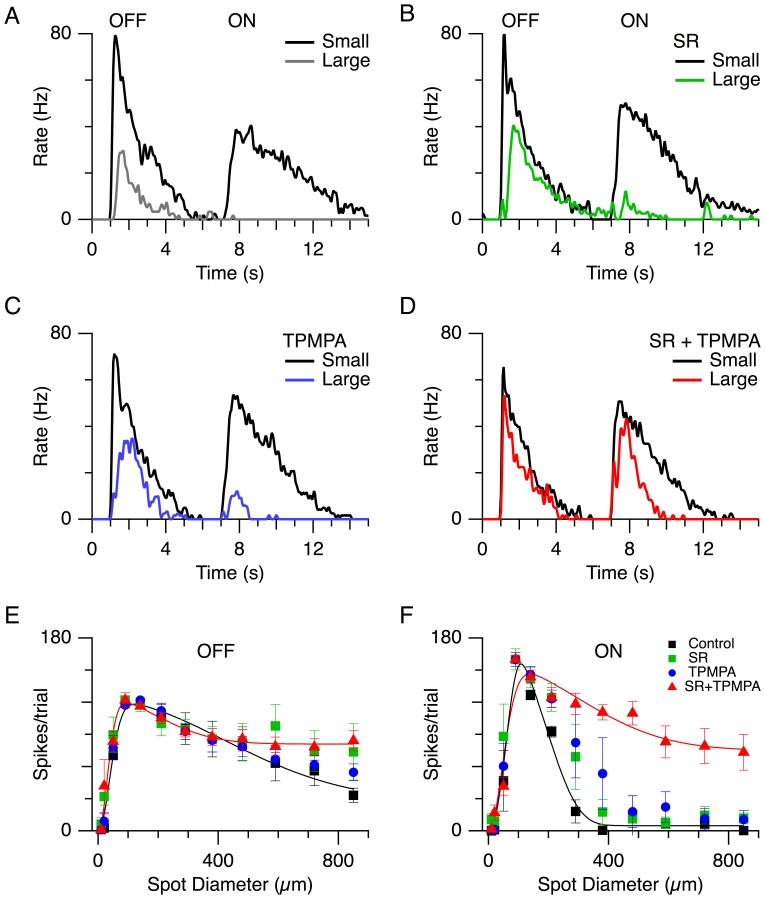
GABAergic inhibition contributes to surround suppression in LEDs. Spike-time histograms averaged from 4 cells, showing responses to a small dark spot (100 µm diameter, dark trace) or large dark spot (850 µm diameter, lighter trace). (A) Control shows that the large spot produced strong surround suppression. Surround suppression is partially relieved in both OFF and ON systems during selective application of 10 µM SR, a GABA_A_ antagonist (B) and 30 µM TPMPA, a GABA_C_ antagonist (C). (D) Surround suppression was relieved when GABAergic transmission was blocked by co-application of SR-95331and 30 µM TPMPA. (E,F) The total number of spikes per trial as a function of the stimulus spot size, for the same 4 cells; the data points (mean ± SD) have been normalized to the average value obtained for the 100 µm spot in control. Control, black; SR, green; TPMPA, blue; SR+TPMA, red.

The centre-activated spike responses (dark spot, 100 µm diameter, Fig. 3A) were suppressed when the stimulus was expanded to activate the centre-plus-surround (850 µm diameter, Fig. 3A), indicating the presence of an antagonistic surround. The OFF response was reduced by about two-thirds while the ON response was completely suppressed. The GABA_A_-receptor antagonist, 10 µM SR 95531 (6-imino-3-(4-methoxyphenyl)-1(6H)-pyridazinebutanoic acid hydrobromide), and the GABA_C_ receptor antagonist, 30 µM TPMPA ((1,2,5,6-tetrahydropyridin-4-yl)methylphosphinic acid), whether applied alone or together, had little effect on the centre-only response (compare black traces Fig. 3A–D). Neither antagonist alone significantly relieved the surround suppression elicited by the largest spot (850 µm) for OFF and ON responses (green trace, Fig. 3B, blue trace Fig. 3C); however, co-application of 10 µM SR and 30 µM TPMPA significantly relieved the surround suppression for the ON response at spot diameters >140 µm (Fig. 3D,F; P<0.029), and the OFF response at a spot diameter of 850 µm (Fig. 3E,F; P = 0.040, n = 4). These results confirm that both GABA_A_ and GABA_C_ receptor activity contributes to the surround antagonism in the LEDs under our recording conditions [8]. The surround remaining for both the ON and OFF responses during GABAergic block presumably reflects activity of the horizontal cells (HCs) in the OPL [24].

**Figure 4:**
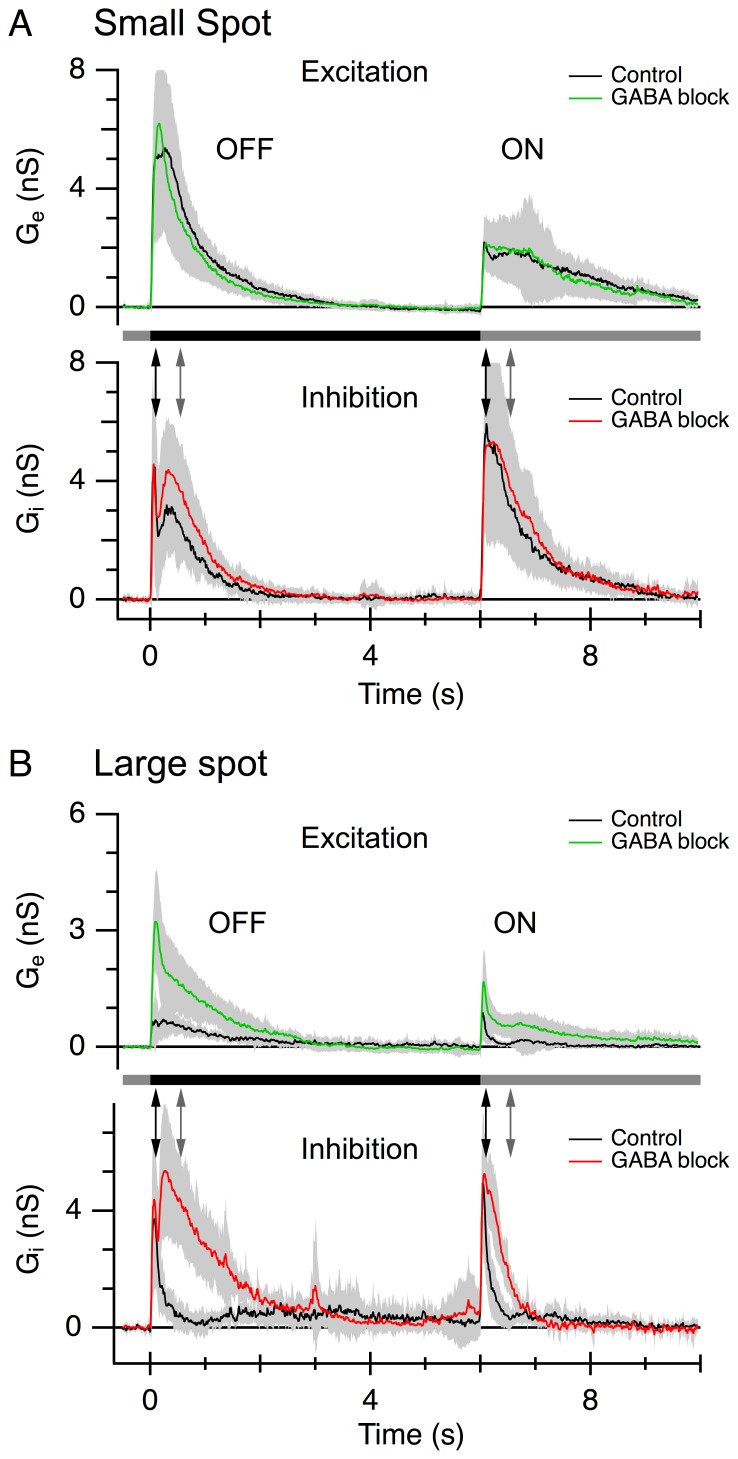
Surround suppression of both excitatory and inhibitory inputs is relieved by GABAergic antagonists. The stimulus was a 100 µm diameter dark spot (A) or a 850 µm diameter dark spot (B) centred in the receptive field; the stimulus timing is shown by the bars. Traces show the average conductances from 7 cells. Control responses are shown in black. The corresponding conductances in the presence of GABAergic blockers (30 µM TPMPA and 10 µM SR-95331) are shown in green for excitation and red for inhibition. Shading shows the standard deviation. The arrows show the early and late time-points used for comparison of the response amplitudes.

Analysis of the excitatory synaptic conductances during centre stimulation and centre-plus-surround stimulation confirms the previous findings that surround inhibition for excitation is largely mediated presynaptically [7], and that it is mediated by GABAergic transmission [8]. Surround suppression was assessed by measuring the amplitude of the conductances in 7 cells at two time-points, 100 ms (early) and 500 ms (late), after onset (OFF-response) and termination of the stimulus (ON-response). The OFF and ON excitatory conductances elicited by a small-field stimulus (100 µm diameter; Fig. 4A, black) were suppressed by a large-field stimulus (850 µm diameter) at both time-points (Fig. 4B, black). At the early and late time-points, the OFF-response was suppressed by 84±4% (P = 0.0037) and 84±3% (P = 0.0014), respectively. The corresponding values for the ON-response were 63±9% (P = 0.0024) and 94±3% (P = 0.0042). Blocking GABAergic transmission (10 µM SR +30 µM TPMPA) reduced surround suppression of excitation, evident as a significant increase in the amplitude of the excitation during large-field stimulation (Fig. 4B, amplitude green>black; OFF: early, P<0.001, late, P = 0.027; ON: early, P = 0.0054, late, P = 0.0065).

We also examined the effect of the GABAergic blockers on the feed-forward inhibition. Surround activation had no consistent effect on the amplitude of the inhibition at the early time-point. The OFF inhibition at the early time-point was 98±21% of control (P = 0.40) and the ON inhibition was 115±26% (P = 0.58). However, the feed-forward inhibition at the late time-point was suppressed by 76±15% (P = 0.019) for the OFF inhibition and 79±13% (P = 0.0081) for the ON inhibition (compare black traces of lower panels in Fig. 4A,B). The GABA blockers relieved the surround suppression of feed-forward inhibition, by significantly increasing the amplitude of the inhibition measured at the late time-point (Fig. 4B, amplitude red>black; OFF, P<0.001, ON, P<0.001). These results indicate that GABAergic pathways mediate suppressive surround inhibition for both excitatory and inhibitory inputs to LEDs, and demonstrate an important role for presynaptic GABAergic mechanisms in the spatial tuning of LEDs. Strychnine alone had little effect on the surround suppression of the excitatory inputs or inhibitory inputs [33](data not shown).

### Effects of Tetrodotoxin (TTX)

**Figure 5:**
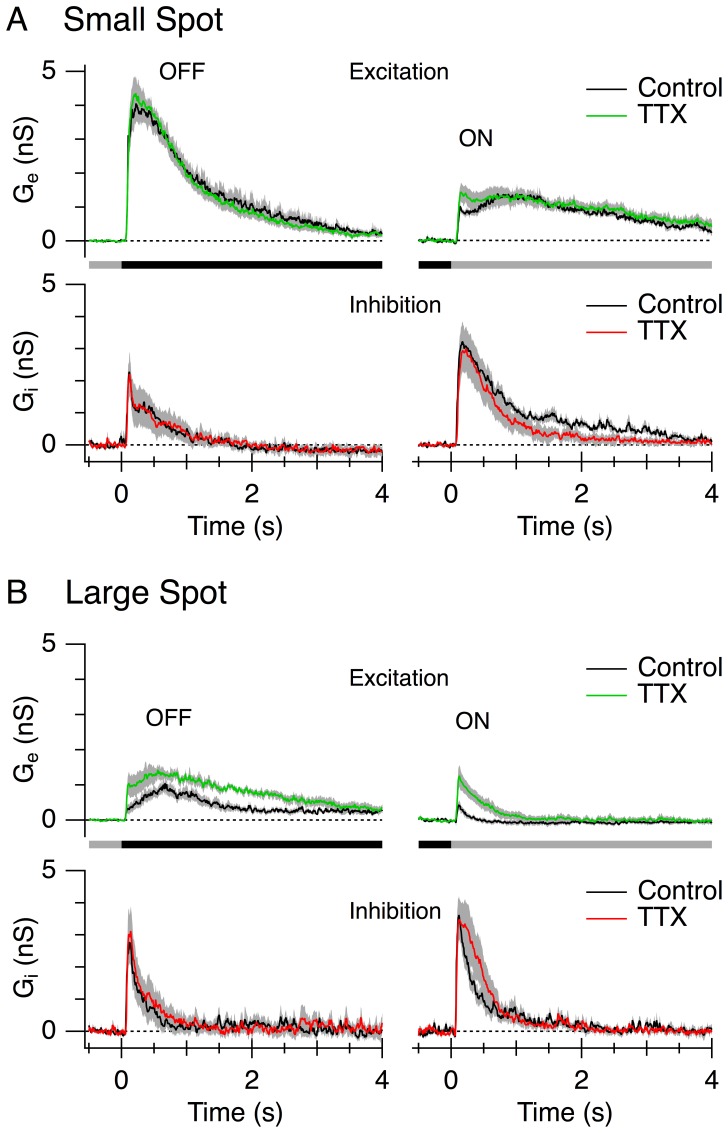
Surround suppression is weakly dependent on TTX-sensitive sodium channels. The stimulus comprised a 100 µm diameter dark spot (A) or a 850 µm diameter dark spot (B) centred in the receptive field; the stimulus timing is shown by the bars. Excitatory (A,B upper panel) and inhibitory (A,B lower panel) conductances were measured under control conditions (black traces) and in the presence of 0.5 µM TTX (coloured traces) for the small and large spots. Each trace represents the average conductance from 6 cells. Shaded region shows SEM.

Previous studies have indicated that the suppressive surround of retinal ganglion cells is partially mediated by amacrine cells that depend on TTX-sensitive sodium channels for signal transmission [6], [34]–[36]. To determine whether this is also the case for LEDs in rabbit, we compared synaptic conductances elicited in the presence and absence of TTX. During centre stimulation, 0.5 µM TTX had no effect on the excitatory or inhibitory conductance (Fig. 5A). During centre-plus-surround stimulation, TTX slightly relieved the surround suppression of the excitatory conductance (Fig. 5B, OFF, P = 0.011, ON, P = 0.012, n = 6), but had no effect on the surround suppression of the inhibition (Fig. 5B). These results raise the possibility that the amacrine cells mediating surround suppression may differ for excitation and inhibition.

## Discussion

The different types of retinal ganglion cells represent distinct spatio-temporal filters that transmit overlapping bands of visual information [37]. Uniquely among ganglion cells, the LEDs are sensitive to high spatial frequencies but low temporal frequencies, and we have shown previously that the spatial and temporal tuning of these cells is dependent on presynaptic inhibition [7]. More recently, another study – also in rabbit retina – showed that the feedback inhibition onto the bipolar cells driving LEDs was GABAergic while the feed-forward inhibition was glycinergic [8]. The results presented here confirm these findings for luminance surrounds, and further demonstrate that GABAergic surround inhibition suppresses the feed-forward glycinergic inhibition. Blocking glycinergic inhibition in the retina produced a significant effect on the delay to the first spike in the LEDs, an effect that was particularly marked for the ON response, which showed the largest delay. This effect can be explained by the strong suppression of the feed-forward inhibition to the LEDs. Thus, although the delay to the activation of the excitatory and inhibitory inputs is similar in control and strychnine, the increased excitatory/inhibitory ratio in the presence of strychnine presumably reduced the spike-delay by allowing the cell to reach spike threshold earlier. Although Russell & Werblin [8] also found that the excitation and inhibition activated with similar delays, they did not observe an effect on the spike delay. Perhaps the higher stimulus contrasts used in that study (–100% and +300%) contributed to this difference.

**Figure 6:**
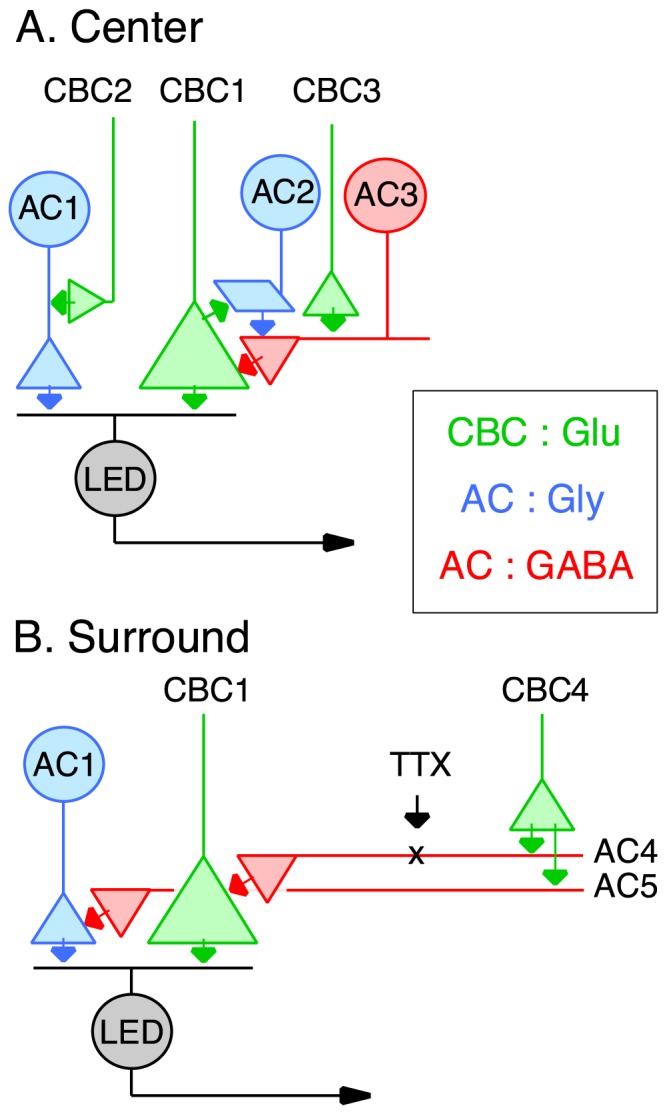
Summary circuit diagrams for synaptic inputs to LED ganglion cells. Similar circuitry is envisaged for the OFF and ON inputs. All cone bipolar cells (CBC, green) in the diagram have the same sign response, i.e. OFF or ON. GABAergic amacrine cells are red, glycinergic amacrine cells are blue. Top: Centre responses are mediated by relatively sustained glutamatergic bipolar cell inputs (CBC1) and di-synaptic feed-forward glycinergic amacrine cell inputs (CBC2→AC1), which are more transient. Note that, despite the additional synapse, these glycinergic inputs activate rapidly enough to veto excitation, and delay spiking in the LED. The suppressive effect of strychnine on the excitatory input to the LED might be accounted for by a cascade of glycinergic and GABAergic synapses. Under control conditions, activation of a glycinergic amacrine cell (AC2) suppresses presynaptic inhibition from a GABAergic amacrine cell (AC3). The resulting dis-inhibition extends glutamate release from CBC1. Application of strychnine blocks this effect, thus allowing presynaptic inhibition from AC3 to suppress the excitatory input to the LED. This model requires that CBC3 drive tonic activity in AC3. Bottom: Surround inhibition is mediated by presynaptic GABAergic inhibition of the excitatory bipolar cells (AC4), and the feed-forward glycinergic amacrine cells (AC5). Different amacrine cells are shown since TTX partially suppresses the excitatory but not the inhibitory surround.

Previous studies have shown that knockout of glycine receptors in the mouse results in a loss of maintained firing and more transient spiking responses in ganglion cells [33], [38]. Such effects on spiking mirror a consistent effect of strychnine on the excitatory inputs to LEDs, which became more transient (e.g. Fig. 1D, 4A, upper panel), and indicate that glycinergic amacrine activity has a dis-inhibitory effect by prolonging glutamate release from bipolar cells, most likely through intervening amacrine cells. Thus glycinergic transmission in the retina contributes to the low temporal tuning of LEDs via two mechanisms (Fig. 6A): 1) feed-forward glycinergic inhibition, which produces longer spike-delays at stimulus onset, and 2) poly-synaptic dis-inhibition that prolongs excitatory inputs.

The temporal tuning via glycinergic feed-forward inhibition was also demonstrated using flicker stimulation. The application of strychnine extended the spiking responses of the LEDs during flicker stimulation, an effect that also appeared to correlate with suppression of direct inhibitory inputs. However, even with glycinergic inhibition blocked, the cells responded to only a few stimulus cycles at the higher frequencies. Analysis of the synaptic conductance indicates that this is likely due to adaptation of the excitatory inputs during maintained flicker, occurring with a time-constant of several seconds (Fig. 2B). We also used ginkgolide to block inhibition from within the recorded cell, thus leaving the presynaptic circuitry intact. These experiments provided further evidence that a major role for postsynaptic glycinergic input is to delay the onset of spiking in the LEDs.

At scotopic and mesopic light levels, when the rod-pathway is active, OFF-bipolar cells are driven by AII amacrine cells, via a glycinergic synapse [39]. Thus the suppression of excitation by strychnine could result from suppression of rod-pathway transmission. Notwithstanding the background intensity used, which should saturate rod photoreceptors, we found that the excitatory OFF conductance at early times (∼120 ms after the step, Fig 1D) in the presence of strychnine equalled or exceeded the value in control. Since flash-responses of dark-adapted rod-bipolar cells reach a peak in about 130 ms [40]–[42], these results indicate that the suppression of excitation during application of glycinergic antagonist is unlikely to result from rod-pathway suppression.

We propose that the glycinergic suppression of ON and OFF excitation might reflect signalling though an intervening, presumably GABAergic, amacrine cell. A synaptic circuit that might account for this effect has tonic GABAergic inhibition onto the bipolar cell terminals that is suppressed through a glycinergic amacrine cell (Fig. 6A). Serial inhibitory networks are expected from previous physiological and anatomical studies [19], [43]–[47]. The mechanisms by which glycinergic transmission modulates the gain of excitatory inputs to LEDs merits further study.

The data confirm previous observations showing that the inhibitory surround that shapes the spatial tuning of the spike output acts presynaptically to the LED [7], [8]. We also confirmed that the spatial extent of the surround differed for the ON and OFF responses, suggesting differences in the underlying circuitry. Application of either GABA_A_ or GABA_C_ antagonists alone did not relieve the surround suppression for either the OFF or ON spiking (Fig. 3B,C). However co-application of GABA_A_ and GABA_C_ antagonists more effectively relieved the surround suppression in both OFF and ON responses (Fig. 3D), suggesting that both GABA_A_ and GABA_C_ pathways contributed to feedback inhibition to the bipolar cells that drive the LED. During GABA block, centre stimulation with a small spot produced a peak glycinergic inhibition that was similar in magnitude to that produced by centre-plus-surround stimulation with a large spot (compare Fig. 4A & B). This indicates that the glycinergic input is maximally activated by the small spot and, therefore, is likely to originate from a small-field amacrine cell. This would be in keeping with a major role in temporal processing, rather than spatial integration. Moreover, the fast transient component of inhibition that is evident during surround stimulation indicates that the bipolar cells driving this residual inhibitory input are not subject to the same surround suppression as the bipolar cells driving the LED centre response (compare black traces Fig. 4B). Together, the results predict that bipolar cells produce two types of excitatory output. 1) Feed-forward excitation that drives the centre responses of LEDs (Fig. 4; [7]) and other ganglion cells [24], [35], [48], and which can be strongly suppressed by surround inhibition (Fig. 6A, CBC1). 2) surround excitation that drives the GABAergic amacrine cells that produce the surround suppression of the feed-forward excitation (Fig. 6B, CBC4). To be effective on the spatial scales that produce presynaptic surround inhibition, the ‘surround’ bipolar cells themselves must not be subject to surround inhibition. Future work will need to determine whether these two forms of excitatory output can occur from different axon terminals of the same bipolar cell, or from different populations of bipolar cells [49].

Previous work has shown that surround inhibition in some GCs is dependent on TTX-sensitive sodium channels, presumably because action-potentials in amacrine cells are required to rapidly convey the inhibitory signals across the retina [6], [34]–[36], [48], [50]. TTX slightly relieved the suppression of the excitatory inputs to LEDs, reflecting a disinhibition of the bipolar cell inputs, but had no obvious effect on the surround suppression of the inhibitory inputs (Fig. 5). This difference suggests that the suppression of excitation and inhibition by the surround might be mediated by distinct types of GABAergic amacrine cells that differ in their TTX sensitivity (Fig. 6B); however, we cannot preclude an effect at the level of the bipolar cells, since TTX-sensitive sodium channels have been reported in a number of mammalian bipolar cell types [51]–[54]. Interestingly, in the mouse retina, TTX completely suppressed surround inhibition in W3 ganglion cells, which are thought to be homologous to the LED cells in the rabbit retina [6]. The weaker effects of TTX on the surround observed here might have been due to differences in the stimuli presented, since the previous analysis of W3 ganglion cells used stimuli that covered much larger surrounding regions, which might have preferentially activated wide-field spiking amacrine cells that integrate and transmit signals over larger distances. It is noteworthy that TTX had little effect on either excitation or inhibition during centre stimulation (Fig. 5A), suggesting that the bipolar cells that provide the excitation, or that drive the inhibitory amacrine cells (Fig. 6A, CBC1, CBC2), do not rely on voltage-gated sodium channels for signal transmission.
